# Crystal structure of *trans*-(1,8-dibutyl-1,3,6,8,10,13-hexa­aza­cyclo­tetra­decane-κ^4^ *N* ^3^,*N* ^6^,*N* ^10^,*N* ^13^)bis­(isonicotinato-κ*O*)copper(II) from synchrotron data

**DOI:** 10.1107/S2056989015001115

**Published:** 2015-01-24

**Authors:** Jong Won Shin, Dae-Woong Kim, Jin Hong Kim, Dohyun Moon

**Affiliations:** a Beamline Department, Pohang Accelerator Laboratory, 80 Jigokro-127-beongil, Nam-Gu Pohang, Gyeongbuk 790-784, South Korea

**Keywords:** Crystal structure, aza­macrocyclic ligand, Jahn–Teller distortion, isonicotinic acid, offset π–π inter­action, hydrogen bonds, synchrotron data

## Abstract

The title compound, [Cu(C_6_H_4_NO_2_)_2_(C_16_H_38_N_6_)] has been synthesized and characterized by structure analysis based on synchrotron data and by FT–IR spectroscopy. The asymmetric unit consists of half of the Cu^II^ complex, the other half being completed by inversion symmetry. The Cu^II^ ion has a tetra­gonally distorted octa­hedral coordination sphere with four secondary N atoms of the aza­macrocyclic ligand in the equatorial plane [Cu—N_eq_ = 2.018 (12) Å] and two O atoms of the isonicotinate anions at the axial positions [Cu—O_ax_ = 2.4100 (11) Å]. Intra­molecular N—H⋯O hydrogen bonds between one of the secondary amine N—H groups of the aza­macrocyclic ligand and the non-coordinating O atom of the isonicotinate ions stabilize the mol­ecular structure. Inter­molecular N—H⋯N hydrogen bonds between the other macrocyclic N—H group and the pyridine N atom of an adjacent isonicotinate anion as well as π–π inter­actions [centroid-to-centroid distance 3.711 (2) Å] lead to the formation of rods parallel to [001].

## Abstract 2

The Cu^II^ ion in the title compound shows a distorted octa­hedral coordination environment defined by four N atoms of the aza­macrocylic ligand in the equatorial plane and two O atoms of the isonicotinate ions in the axial sites. In the crystal, the mol­ecules are connected by hydrogen bonds and π–π inter­actions, forming rods parallel to [001].

## Chemical context   

The coordination chemistry of macrocyclic ligands has attracted extensive inter­est due to their potential applications in material science, chemistry and metalloenzymes (Lehn, 1995 ▸; Carnes *et al.*, 2014 ▸). In particular, Cu^II^ macrocylic complexes involving vacant sites in an axial position are feasible candidates for assembling supra­molecular materials, with potential applications as gas-storage materials (Suh *et al.*, 2012 ▸) as well as catalysts for co-polymerization of carbon dioxide and cyclo­hexene oxide (Tsai *et al.*, 2014 ▸). Moreover, Cu^II^ complexes with tetra­aza­macrocyclic ligands involving alkyl moieties have been investigated as magnetic materials with various auxiliary ligands such as metal cyanide, azide, and dicyanamide (Bi *et al.*, 2012 ▸).

Isonicotinic acid is a versatile anion which can easily bind to transition metals *via* the carboxyl group or the pyridine N atom, thus allowing the assembly of multidimensionally structured compounds or heterometallic complexes (Liu *et al.*, 2006 ▸).

Here, we report on the synthesis and crystal structure of a Cu^II^ aza­macrocyclic complex with two isonicotinato co-ligands, *trans*-(1,8-dibutyl-1,3,6,8,10,13-hexa­aza­cyclo­tetra­decane-κ^4^ *N* ^3^ *,N* ^6^ *,N* ^10^ *,N* ^13^)bis­(isonicotinato-κ*O*)copper(II), (I).
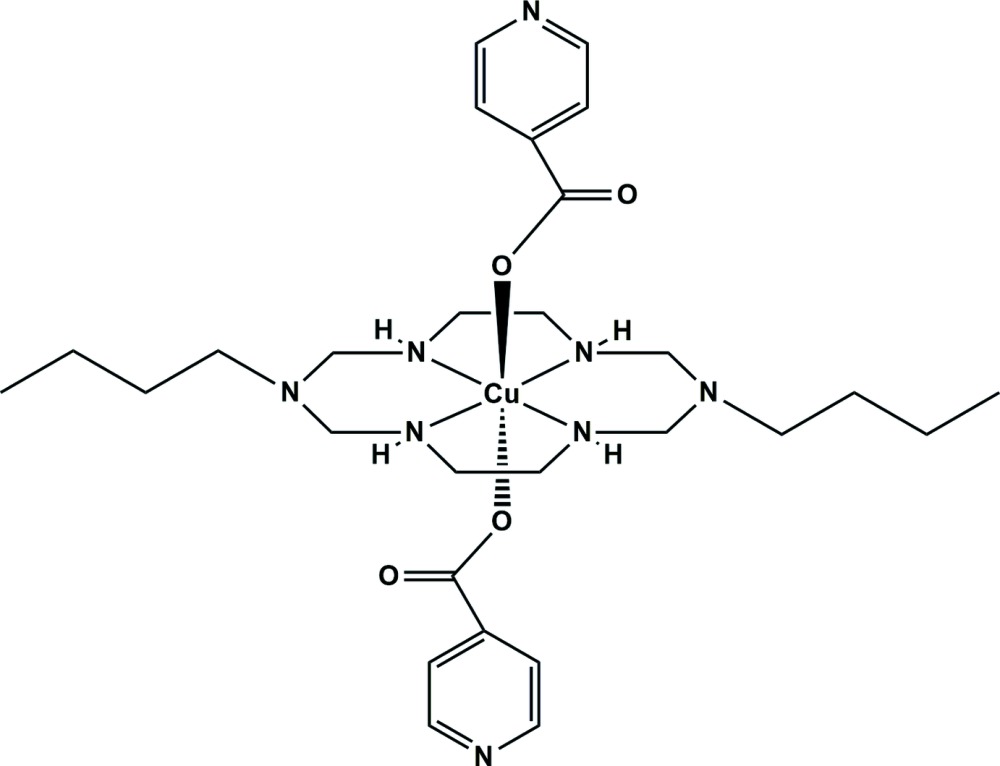


## Structural commentary   

**Figure 1:**
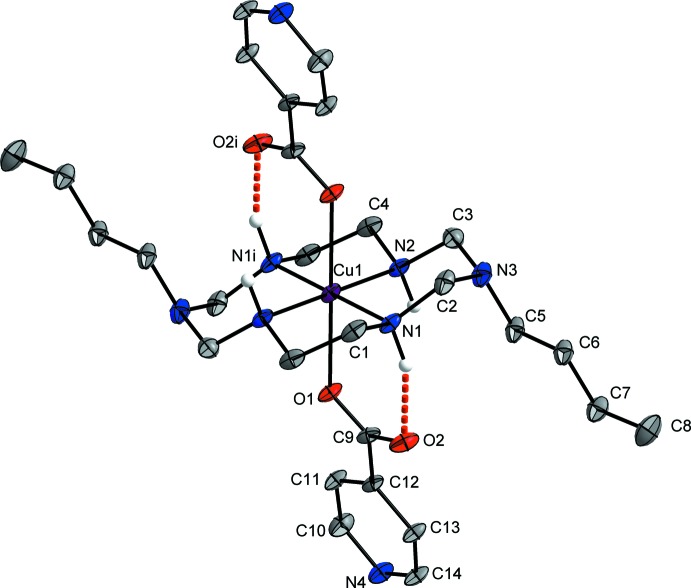
The mol­ecular structure of (I), showing the atom-labelling scheme, with displacement ellipsoids drawn at the 50% probability level. Intra­molecular N—H⋯O hydrogen bonds are shown as red dashed lines. [Symmetry code: (i) −*x* + 1, −*y* + 1, −*z* + 1.]

In compound (I), the Cu^II^ ion lies on an inversion center and is coordinated by the four secondary amine N atoms of the aza­macrocyclic ligand in the equatorial plane and by two O atoms from the isonicotinate anions at the axial positions, resulting in a tetra­gonally distorted octa­hedral geometry, as shown in Fig. 1 ▸. The average Cu—N_eq_ bond length is 2.018 (12) and the Cu—O_ax_ bond length is 2.4100 (11) Å. This difference can be attributed either to a large Jahn–Teller distortion effect of the Cu^II^ ion and/or to a ring contraction of the aza­macrocyclic ligand (Halcrow, 2013 ▸). The six-membered chelate ring (Cu1–N1–C2–N3–C3–N2) adopts a chair conformation and the five-membered chelate ring (Cu1–N1–C1–C4–N2) a *gauche* conformation (Min & Suh, 2001 ▸). The two C—O bond lengths of the carboxyl­ate group are 1.255 (2) and 1.258 (2) Å, indicating that this group is fully delocalized with a bond angle (O1—C9—O2) of 126.8 (1)°. Intra­molecular N1—H1⋯O2 hydrogen bonds between one of the secondary amine groups of the aza­macrocyclic ligand and the O atoms of a coordinating isonicotinate anion stabilize the mol­ecular structure (Fig. 1 ▸ and Table 1 ▸).

## Supra­molecular features   

**Figure 2:**
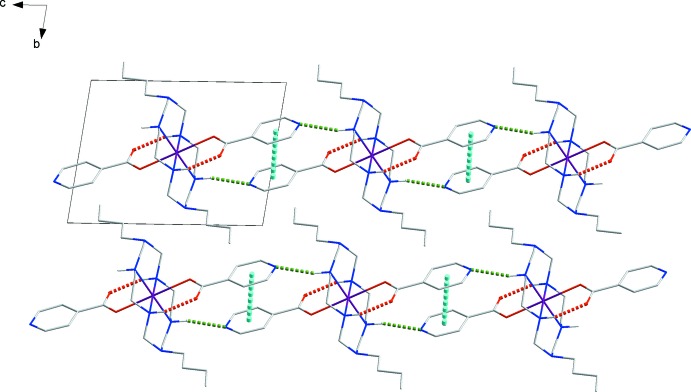
View of the crystal packing of (I), with N—H⋯O hydrogen bonds and π–π inter­actions shown as dashed lines (red: intra­molecular hydrogen bonds, green: inter­molecular hydrogen bonds, cyan: π–π inter­actions).

The N atoms of the isonicotinate ions form inter­molecular N2—H2⋯N4 hydrogen bonds (Steed & Atwood, 2009 ▸) with the adjacent secondary amine group of the aza­macrocyclic ligand (Fig. 2 ▸ and Table 1 ▸). The pyridine rings of the iso­nico­tinate co-ligand are involved in π–π stacking inter­actions [centroid-to-centroid distance 3.711 (2) Å]. The inter­planar separation and dihedral angle between the pyridine rings in adjacent isonicotinate anions are 3.522 (2) Å and 0.0°, respectively, implying a parallel assignment to each other (Hunter & Sanders, 1990 ▸). The hydrogen-bonding and π–π inter­actions generate rods of inter­acting mol­ecules parallel to [001].

## Database survey   

A search of the Cambridge Structural Database (Version 5.35, May 2014 with three updates; Groom & Allen, 2014 ▸) indicate that only one Cu^II^ aza­macrocyclic complex having butyl pendant groups has been reported (Kim *et al.*, 2015 ▸).

## Synthesis and crystallization   

Compound (I) was prepared as follows. The starting complex, [Cu(C_16_H_38_N_6_)(ClO_4_)_2_], was obtained by a slight modification of the reported method (Kim *et al.*, 2015 ▸). To an MeCN (10 mL) solution of [Cu(C_16_H_38_N_6_)(ClO_4_)_2_] (0.15 g, 0.26 mmol) was slowly added an MeCN solution (5 mL) containing iso­nicotinic acid (0.064 g, 0.52 mmol) and excess tri­ethyl­amine (0.06 g, 0.60 mmol) at room temperature. The formed purple precipitate was filtered off, washed with MeCN, and diethyl ether, and dried in air. Single crystals of the title compound were obtained by layering a MeCN solution of isonicotinic acid on the MeCN solution of [Cu(C_16_H_38_N_6_)(ClO_4_)_2_] for several days. Yield: 0.087 g (54%). FT–IR (ATR, cm^−1^): 3197, 3097, 2954, 2929, 1596, 1544, 1365, 1280, 1016, 964.

**Safety note:** Although we have experienced no problem with the compounds involved in this study, perchlorate salts of metal complexes are often explosive and should be handled with great caution.

## Refinement   

Crystal data, data collection and structure refinement details are summarized in Table 2 ▸. All H atoms were placed in geometrically idealized positions and constrained to ride on their parent atoms, with C—H distances of 0.95 (ring H atoms) or 0.98–0.99 Å (open-chain H atoms) and an N—H distance of 1.0 Å with *U*_iso_(H) values of 1.2 or 1.5*U*_eq_ of the parent atoms.

## Supplementary Material

Crystal structure: contains datablock(s) I. DOI: 10.1107/S2056989015001115/wm5115sup1.cif

Structure factors: contains datablock(s) I. DOI: 10.1107/S2056989015001115/wm5115Isup2.hkl

CCDC reference: 1044260

Additional supporting information:  crystallographic information; 3D view; checkCIF report

## Figures and Tables

**Table 1:** Hydrogen-bond geometry (, )

*D*H*A*	*D*H	H*A*	*D* *A*	*D*H*A*
N1H1O2	1.00	1.98	2.9179(16)	155
N2H2N4^i^	1.00	2.21	3.1160(16)	150

Symmetry code: (i) 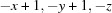.

**Table 2:** Experimental details

Crystal data	
Chemical formula	[Cu(C_6_H_4_NO_2_)_2_(C_16_H_38_N_6_)]
*M*_r_	622.27
Crystal system, space group	Triclinic, *P* 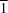
Temperature (K)	100
*a*, *b*, *c* ()	8.0490(16), 8.3000(17), 11.175(2)
, , ()	81.16(3), 87.14(3), 86.68(3)
*V* (^3^)	735.8(3)
*Z*	1
Radiation type	Synchrotron, = 0.630
(mm^1^)	0.57
Crystal size (mm)	0.08 0.03 0.03

Data collection	
Diffractometer	ADSC Q210 CCD area detector
Absorption correction	Empirical (using intensity measurements) (*HKL3000sm *SCALEPACK**; Otwinowski Minor, 1997 ▸)
*T*_min_, *T*_max_	0.958, 0.983
No. of measured, independent and observed [*I* > 2(*I*)] reflections	7574, 3882, 3608
*R*_int_	0.018
(sin /)_max_ (^1^)	0.696

Refinement	
*R*[*F* ^2^ > 2(*F* ^2^)], *wR*(*F* ^2^), *S*	0.030, 0.086, 1.09
No. of reflections	3882
No. of parameters	188
H-atom treatment	H-atom parameters constrained
_max_, _min_ (e ^3^)	0.43, 0.62

Computer programs: *PAL ADSC Quantum-210 ADX* (Arvai Nielsen, 1983 ▸), *HKL3000sm* (Otwinowski Minor, 1997 ▸), *SHELXT2014/5* (Sheldrick, 2015*a* ▸), *SHELXL2014/7* (Sheldrick, 2008 ▸, 2015*b* ▸), *DIAMOND4* (Putz Brandenburg, 2014 ▸) and *publCIF* (Westrip, 2010 ▸).

## supplementary crystallographic information

### Crystal data

**Table:**

[Cu(C_6_H_4_NO_2_)_2_(C_16_H_38_N_6_)]	*Z* = 1
*M**_r_* = 622.27	*F*(000) = 331
Triclinic, *P*1	*D*_x_ = 1.404 Mg m^−^^3^
*a* = 8.0490 (16) Å	Synchrotron radiation, λ = 0.630 Å
*b* = 8.3000 (17) Å	Cell parameters from 21514 reflections
*c* = 11.175 (2) Å	θ = 0.4–33.6°
α = 81.16 (3)°	µ = 0.57 mm^−^^1^
β = 87.14 (3)°	*T* = 100 K
γ = 86.68 (3)°	Needle, purple
*V* = 735.8 (3) Å^3^	0.08 × 0.03 × 0.03 mm

### Data collection

**Table:**

ADSC Q210 CCD area detector diffractometer	3608 reflections with *I* > 2σ(*I*)
Radiation source: PLSII 2D bending magnet	*R*_int_ = 0.018
ω scan	θ_max_ = 26.0°, θ_min_ = 2.7°
Absorption correction: empirical (using intensity measurements) (*HKL3000sm *SCALEPACK**; Otwinowski & Minor, 1997)	*h* = −11→11
*T*_min_ = 0.958, *T*_max_ = 0.983	*k* = −11→11
7574 measured reflections	*l* = −15→15
3882 independent reflections	

### Refinement

**Table:**

Refinement on *F*^2^	0 restraints
Least-squares matrix: full	Hydrogen site location: inferred from neighbouring sites
*R*[*F*^2^ > 2σ(*F*^2^)] = 0.030	H-atom parameters constrained
*wR*(*F*^2^) = 0.086	*w* = 1/[σ^2^(*F*_o_^2^) + (0.051*P*)^2^ + 0.1939*P*] where *P* = (*F*_o_^2^ + 2*F*_c_^2^)/3
*S* = 1.09	(Δ/σ)_max_ < 0.001
3882 reflections	Δρ_max_ = 0.43 e Å^−^^3^
188 parameters	Δρ_min_ = −0.62 e Å^−^^3^

### Special details

**Table:**

Geometry. All e.s.d.'s (except the e.s.d. in the dihedral angle between two l.s. planes) are estimated using the full covariance matrix. The cell e.s.d.'s are taken into account individually in the estimation of e.s.d.'s in distances, angles and torsion angles; correlations between e.s.d.'s in cell parameters are only used when they are defined by crystal symmetry. An approximate (isotropic) treatment of cell e.s.d.'s is used for estimating e.s.d.'s involving l.s. planes.

### Fractional atomic coordinates and isotropic or equivalent isotropic displacement parameters (Å^2^)

**Table:**

	*x*	*y*	*z*	*U*_iso_*/*U*_eq_	
Cu1	0.5000	0.5000	0.5000	0.01489 (8)	
O1	0.43502 (12)	0.38249 (14)	0.32476 (8)	0.0228 (2)	
O2	0.19078 (12)	0.51588 (15)	0.27708 (9)	0.0250 (2)	
N1	0.27675 (13)	0.62129 (14)	0.50255 (9)	0.0174 (2)	
H1	0.2211	0.6111	0.4262	0.021*	
N2	0.60724 (13)	0.68104 (14)	0.38483 (9)	0.0174 (2)	
H2	0.5757	0.6720	0.3008	0.021*	
N3	0.37958 (16)	0.88517 (15)	0.40936 (10)	0.0241 (2)	
N4	0.36803 (15)	0.28785 (16)	−0.10314 (10)	0.0234 (2)	
C1	0.17805 (15)	0.53580 (19)	0.60560 (11)	0.0208 (3)	
H1A	0.0580	0.5652	0.5960	0.025*	
H1B	0.2109	0.5678	0.6825	0.025*	
C2	0.28664 (18)	0.79685 (18)	0.50926 (11)	0.0231 (3)	
H2A	0.1722	0.8471	0.5123	0.028*	
H2B	0.3389	0.8084	0.5856	0.028*	
C3	0.55623 (18)	0.84773 (18)	0.40891 (12)	0.0239 (3)	
H3A	0.5973	0.8617	0.4885	0.029*	
H3B	0.6110	0.9276	0.3466	0.029*	
C4	0.78914 (16)	0.64587 (18)	0.39220 (11)	0.0211 (3)	
H4A	0.8291	0.6792	0.4666	0.025*	
H4B	0.8484	0.7070	0.3212	0.025*	
C5	0.30584 (19)	0.90191 (18)	0.29031 (12)	0.0241 (3)	
H5A	0.2879	0.7920	0.2709	0.029*	
H5B	0.3848	0.9552	0.2279	0.029*	
C6	0.13998 (19)	1.00201 (18)	0.28563 (12)	0.0241 (3)	
H6A	0.0599	0.9481	0.3470	0.029*	
H6B	0.1572	1.1119	0.3056	0.029*	
C7	0.06801 (19)	1.0188 (2)	0.16070 (13)	0.0270 (3)	
H7A	0.0460	0.9091	0.1425	0.032*	
H7B	0.1505	1.0676	0.0990	0.032*	
C8	−0.0932 (2)	1.1251 (2)	0.15327 (15)	0.0373 (4)	
H8A	−0.1767	1.0745	0.2119	0.056*	
H8B	−0.1347	1.1357	0.0713	0.056*	
H8C	−0.0720	1.2335	0.1719	0.056*	
C9	0.31897 (15)	0.43043 (17)	0.25450 (10)	0.0177 (2)	
C10	0.48401 (17)	0.2439 (2)	−0.02071 (12)	0.0246 (3)	
H10	0.5792	0.1802	−0.0429	0.029*	
C11	0.47339 (16)	0.28583 (18)	0.09515 (11)	0.0204 (3)	
H11	0.5587	0.2502	0.1504	0.024*	
C12	0.33664 (14)	0.38038 (16)	0.12895 (10)	0.0157 (2)	
C13	0.21551 (16)	0.42803 (18)	0.04430 (11)	0.0198 (2)	
H13	0.1201	0.4935	0.0636	0.024*	
C14	0.23614 (17)	0.37841 (19)	−0.06890 (11)	0.0223 (3)	
H14	0.1517	0.4106	−0.1254	0.027*	

### Atomic displacement parameters (Å^2^)

**Table:**

	*U*^11^	*U*^22^	*U*^33^	*U*^12^	*U*^13^	*U*^23^
Cu1	0.01176 (11)	0.02337 (13)	0.00843 (10)	0.00026 (7)	0.00131 (6)	0.00020 (7)
O1	0.0233 (5)	0.0349 (6)	0.0105 (4)	0.0010 (4)	−0.0053 (3)	−0.0035 (4)
O2	0.0172 (4)	0.0447 (6)	0.0148 (4)	0.0016 (4)	0.0002 (3)	−0.0113 (4)
N1	0.0156 (5)	0.0287 (6)	0.0070 (4)	0.0019 (4)	0.0007 (3)	−0.0012 (4)
N2	0.0161 (5)	0.0262 (6)	0.0094 (4)	−0.0021 (4)	0.0000 (3)	−0.0002 (4)
N3	0.0318 (6)	0.0249 (6)	0.0143 (5)	0.0043 (5)	−0.0003 (4)	−0.0009 (4)
N4	0.0251 (6)	0.0351 (7)	0.0098 (4)	0.0011 (5)	0.0002 (4)	−0.0042 (4)
C1	0.0136 (5)	0.0381 (7)	0.0095 (5)	0.0004 (5)	0.0026 (4)	−0.0016 (5)
C2	0.0297 (7)	0.0266 (7)	0.0116 (5)	0.0075 (5)	0.0013 (5)	−0.0026 (5)
C3	0.0306 (7)	0.0231 (6)	0.0182 (6)	−0.0035 (5)	−0.0010 (5)	−0.0029 (5)
C4	0.0154 (5)	0.0353 (7)	0.0120 (5)	−0.0057 (5)	0.0016 (4)	−0.0009 (5)
C5	0.0326 (7)	0.0241 (7)	0.0136 (5)	0.0050 (5)	0.0005 (5)	0.0003 (5)
C6	0.0310 (7)	0.0232 (6)	0.0166 (6)	0.0032 (5)	0.0005 (5)	−0.0004 (5)
C7	0.0299 (7)	0.0314 (7)	0.0182 (6)	0.0027 (6)	−0.0001 (5)	−0.0010 (5)
C8	0.0307 (8)	0.0520 (10)	0.0250 (7)	0.0082 (7)	0.0013 (6)	0.0032 (7)
C9	0.0163 (5)	0.0290 (7)	0.0080 (5)	−0.0057 (4)	0.0003 (4)	−0.0022 (4)
C10	0.0233 (6)	0.0365 (8)	0.0135 (5)	0.0061 (5)	0.0009 (5)	−0.0057 (5)
C11	0.0186 (6)	0.0314 (7)	0.0104 (5)	0.0025 (5)	−0.0020 (4)	−0.0014 (5)
C12	0.0146 (5)	0.0254 (6)	0.0069 (4)	−0.0034 (4)	0.0005 (4)	−0.0007 (4)
C13	0.0170 (5)	0.0326 (7)	0.0094 (5)	0.0023 (5)	−0.0008 (4)	−0.0027 (5)
C14	0.0216 (6)	0.0362 (7)	0.0088 (5)	0.0015 (5)	−0.0031 (4)	−0.0029 (5)

### Geometric parameters (Å, º)

**Table:**

Cu1—N1^i^	2.0093 (12)	C3—H3B	0.9900
Cu1—N1	2.0093 (12)	C4—C1^i^	1.512 (2)
Cu1—N2	2.0260 (13)	C4—H4A	0.9900
Cu1—N2^i^	2.0261 (13)	C4—H4B	0.9900
Cu1—O1^i^	2.4100 (11)	C5—C6	1.530 (2)
Cu1—O1	2.4100 (11)	C5—H5A	0.9900
O1—C9	1.2576 (16)	C5—H5B	0.9900
O2—C9	1.2551 (17)	C6—C7	1.522 (2)
N1—C2	1.4775 (19)	C6—H6A	0.9900
N1—C1	1.4778 (16)	C6—H6B	0.9900
N1—H1	1.0000	C7—C8	1.525 (2)
N2—C4	1.4801 (16)	C7—H7A	0.9900
N2—C3	1.4807 (19)	C7—H7B	0.9900
N2—H2	1.0000	C8—H8A	0.9800
N3—C3	1.4377 (19)	C8—H8B	0.9800
N3—C2	1.4394 (19)	C8—H8C	0.9800
N3—C5	1.4676 (18)	C9—C12	1.5211 (17)
N4—C14	1.3393 (18)	C10—C11	1.3888 (18)
N4—C10	1.3402 (18)	C10—H10	0.9500
C1—C4^i^	1.512 (2)	C11—C12	1.3855 (18)
C1—H1A	0.9900	C11—H11	0.9500
C1—H1B	0.9900	C12—C13	1.3908 (16)
C2—H2A	0.9900	C13—C14	1.3887 (17)
C2—H2B	0.9900	C13—H13	0.9500
C3—H3A	0.9900	C14—H14	0.9500
			
N1^i^—Cu1—N1	180.0	H3A—C3—H3B	107.6
N1^i^—Cu1—N2	86.38 (5)	N2—C4—C1^i^	107.66 (11)
N1—Cu1—N2	93.62 (5)	N2—C4—H4A	110.2
N1^i^—Cu1—N2^i^	93.62 (5)	C1^i^—C4—H4A	110.2
N1—Cu1—N2^i^	86.38 (5)	N2—C4—H4B	110.2
N2—Cu1—N2^i^	180.00 (5)	C1^i^—C4—H4B	110.2
N1^i^—Cu1—O1^i^	91.88 (5)	H4A—C4—H4B	108.5
N1—Cu1—O1^i^	88.12 (5)	N3—C5—C6	112.45 (12)
N2—Cu1—O1^i^	92.34 (4)	N3—C5—H5A	109.1
N2^i^—Cu1—O1^i^	87.66 (4)	C6—C5—H5A	109.1
N1^i^—Cu1—O1	88.12 (5)	N3—C5—H5B	109.1
N1—Cu1—O1	91.88 (5)	C6—C5—H5B	109.1
N2—Cu1—O1	87.66 (4)	H5A—C5—H5B	107.8
N2^i^—Cu1—O1	92.34 (4)	C7—C6—C5	111.11 (12)
O1^i^—Cu1—O1	180.0	C7—C6—H6A	109.4
C9—O1—Cu1	126.16 (9)	C5—C6—H6A	109.4
C2—N1—C1	112.34 (10)	C7—C6—H6B	109.4
C2—N1—Cu1	113.64 (9)	C5—C6—H6B	109.4
C1—N1—Cu1	106.52 (8)	H6A—C6—H6B	108.0
C2—N1—H1	108.0	C6—C7—C8	111.54 (13)
C1—N1—H1	108.0	C6—C7—H7A	109.3
Cu1—N1—H1	108.0	C8—C7—H7A	109.3
C4—N2—C3	112.40 (11)	C6—C7—H7B	109.3
C4—N2—Cu1	105.83 (8)	C8—C7—H7B	109.3
C3—N2—Cu1	114.38 (8)	H7A—C7—H7B	108.0
C4—N2—H2	108.0	C7—C8—H8A	109.5
C3—N2—H2	108.0	C7—C8—H8B	109.5
Cu1—N2—H2	108.0	H8A—C8—H8B	109.5
C3—N3—C2	114.61 (11)	C7—C8—H8C	109.5
C3—N3—C5	114.92 (11)	H8A—C8—H8C	109.5
C2—N3—C5	116.23 (12)	H8B—C8—H8C	109.5
C14—N4—C10	116.32 (12)	O2—C9—O1	126.75 (12)
N1—C1—C4^i^	108.03 (10)	O2—C9—C12	116.76 (11)
N1—C1—H1A	110.1	O1—C9—C12	116.49 (11)
C4^i^—C1—H1A	110.1	N4—C10—C11	123.95 (13)
N1—C1—H1B	110.1	N4—C10—H10	118.0
C4^i^—C1—H1B	110.1	C11—C10—H10	118.0
H1A—C1—H1B	108.4	C12—C11—C10	119.03 (12)
N3—C2—N1	114.08 (11)	C12—C11—H11	120.5
N3—C2—H2A	108.7	C10—C11—H11	120.5
N1—C2—H2A	108.7	C11—C12—C13	117.81 (11)
N3—C2—H2B	108.7	C11—C12—C9	121.25 (11)
N1—C2—H2B	108.7	C13—C12—C9	120.94 (11)
H2A—C2—H2B	107.6	C14—C13—C12	118.98 (12)
N3—C3—N2	114.67 (12)	C14—C13—H13	120.5
N3—C3—H3A	108.6	C12—C13—H13	120.5
N2—C3—H3A	108.6	N4—C14—C13	123.90 (12)
N3—C3—H3B	108.6	N4—C14—H14	118.1
N2—C3—H3B	108.6	C13—C14—H14	118.1
			
C2—N1—C1—C4^i^	−166.01 (10)	C5—C6—C7—C8	−177.30 (14)
Cu1—N1—C1—C4^i^	−40.98 (11)	Cu1—O1—C9—O2	−20.4 (2)
C3—N3—C2—N1	−70.73 (16)	Cu1—O1—C9—C12	159.19 (8)
C5—N3—C2—N1	67.19 (15)	C14—N4—C10—C11	0.4 (2)
C1—N1—C2—N3	−179.05 (10)	N4—C10—C11—C12	−0.8 (2)
Cu1—N1—C2—N3	59.93 (13)	C10—C11—C12—C13	0.3 (2)
C2—N3—C3—N2	68.49 (15)	C10—C11—C12—C9	−179.76 (13)
C5—N3—C3—N2	−69.99 (15)	O2—C9—C12—C11	−179.88 (13)
C4—N2—C3—N3	−176.79 (10)	O1—C9—C12—C11	0.48 (19)
Cu1—N2—C3—N3	−56.07 (13)	O2—C9—C12—C13	0.02 (19)
C3—N2—C4—C1^i^	167.50 (10)	O1—C9—C12—C13	−179.62 (12)
Cu1—N2—C4—C1^i^	41.99 (10)	C11—C12—C13—C14	0.4 (2)
C3—N3—C5—C6	−158.17 (12)	C9—C12—C13—C14	−179.48 (12)
C2—N3—C5—C6	64.03 (16)	C10—N4—C14—C13	0.4 (2)
N3—C5—C6—C7	179.31 (12)	C12—C13—C14—N4	−0.8 (2)

Symmetry code: (i) −*x*+1, −*y*+1, −*z*+1.

### Hydrogen-bond geometry (Å, º)

**Table:**

*D*—H···*A*	*D*—H	H···*A*	*D*···*A*	*D*—H···*A*
N1—H1···O2	1.00	1.98	2.9179 (16)	155
N2—H2···N4^ii^	1.00	2.21	3.1160 (16)	150

Symmetry code: (ii) −*x*+1, −*y*+1, −*z*.
